# FilaminA and Formin2 regulate skeletal, muscular, and intestinal formation through mesenchymal progenitor proliferation

**DOI:** 10.1371/journal.pone.0189285

**Published:** 2017-12-14

**Authors:** Gewei Lian, Sneha Kanaujia, Timothy Wong, Volney Sheen

**Affiliations:** Department of Neurology, Beth Israel Deaconess Medical Center and Harvard Medical School, Boston, MA, United States of America; Laboratoire de Biologie du Développement de Villefranche-sur-Mer, FRANCE

## Abstract

The effects of actin dependent molecular mechanisms in coordinating cellular proliferation, migration and differentiation during embryogenesis are not well-understood. We have previously shown that actin-binding Filamin A (FlnA) and actin-nucleating Formin 2 (Fmn2) influence the development of the brain causing microcephaly in mice. In this study, we broaden this phenotype to explore the effects of these two proteins in the development of extra-CNS organ systems, including the gut, muscle, and skeleton. We observed defects in rib and sternum midline closure leading to thoracoabdominal schisis in FlnA+Fmn2 knockout mice, reminiscent of the pentalogy of Cantrell syndrome. These mice exhibit shortened guts, as well as thinned thoracic muscle mass. Immunostaining showed these changes are partially caused by a decrease in the number of presumptive mesenchymal proliferating cells with loss of either FlnA or FlnA+Fmn2. This proliferation defect appears to be in part due to delayed differentiation in these regions. While both FlnA and FlnA+Fmn2 mice show reduced cell death relative to WT control, increased caspase staining was seen in the double null relative to FlnA null suggesting that this could also contribute to the FlnA+Fmn2 phenotype. Therefore FlnA and Fmn2 are likely essential to cell proliferation, differentiation and cell death in a variety of tissues and organs, further reiterating the importance of vesicle trafficking in regulation of development.

## Introduction

Pentalogy of Cantrell (PoC, thoraco-abdominal syndrome) is a rare congenital disorder characterized by a combination of birth defects involving the sternum, diaphragm, pericardium, abdominal wall, and heart [1]. The disorder occurs with varying degrees of severity, but in its most severe form is associated with ectopia cordis (displacement of the heart outside the thoracic cavity) and omphalocele (displacement of abdominal organs outside the abdominal wall). The exact cause of PoC is unknown with most cases believed to occur sporadically from genetic anomalies. Other presentations have been associated with riboflavin deficiency and *Hoxb4* gene disruption in mice [2,3].

Filamin A (FlnA) is an actin-binding protein that is thought to serve as a molecular scaffold to transduce various receptor and intracellular signals through the actin cytoskeleton [4]. While human mutations in FlnA are more commonly associated with a malformation of cortical development, periventricular heterotopia [5], dysregulation of this actin associated protein also leads to skeletal defects, cardiovascular anomalies and gastrointestinal issues [6–8]. Moreover, FlnA is broadly expressed in various cell types and organ systems. These observations suggest a broad role for these actin binding proteins in development.

We have previously shown that FlnA physically interacts with the actin-nucleating protein Formin2 (Fmn2) and that these proteins work in a coordinated fashion to regulate vesicle trafficking and endocytosis [9]. Loss of both FlnA and Fmn2 result in impaired canonical Wnt signaling within neural progenitors by disrupting the trafficking of the Lrp6 receptor, thereby causing microcephaly (small brain) [9]. To address the potential role for these actin associated proteins in regulating development of other organ systems, we characterized the skeletal development in the FlnA and Fmn2 double null mice. Loss of function of these proteins causes many features characteristic of PoC with thoracoabdominal schisis. These phenoytpes are in part due to a reduction in presumptive mesenchymal progenitor proliferation, and not an increase in apoptotic cell death.

## Materials and methods

### Mice

All mouse studies were performed with approval from the Institutional Animal Care and Use Committees (IACUC) of Harvard Medical School and Beth Israel Deaconess Medical Center in accordance with The National Institutes of Health Guide for the Care and Use of Laboratory Animals. Micewere maintained in a pathogen-free facility with a 12 hours light/dark cycle. Mice were fed a standard (42% of total calories from fat; 0.15% cholesterol; Harlan Teklad, USA).

The Dilp2 (null FlnA) mouse strain was obtained from the Comparative and Developmental Genetics Dept. MRC Human Genetics Unit, Edinburgh EH4 2XU, UK. Generation of Fmn2 knockout and Fmn2-GFP transgenic mice were obtained from Dr. Philip Leder in the Department of Genetics, Harvard Medical School, Boston, MA.

Genotyping of the Fmn2 and FlnA mice was previously described [4,10]. The wild-type Fmn2 allele was detected by PCR amplification using the primer pair 5′-CGATGTGAAGTCTGAAGGACAGGC and 5′-AGGTGGTGGTAGTTGTGATGCACTC and the knockout Fmn2 allele by using 5′-CGATGTGAAGTCTGAAGGACAGGC and 5′- GCCGGAGAACCTGCGTGCAATCC. The Dilp2 mouse strain was transferred from C3H to 129/SvJ strain by breeding back with male of 129/SvJ strain. The Dilp2 mice harbor a T to A point mutation in the FlnA gene, which could be identified by PCR and DNA sequencing of the PCR product. The primer sequences for genotyping PCR are: forward primer 5’-GCAGGCATTTTGCTTGTTATTCC and reverse primer 5-ACCTACC- TGTGACACCACCTTCC. Null FlnA and Fmn2 embryos were obtained by breeding FlnA heterozygous+ Fmn2 heterozygous females with FlnA WT+ Fmn2 heterozygous or null males. The null FlnA+Fmn2 mice are embryonic lethal. Pregnant dams were euthanized using ketamine/xylazine followed by cervical dislocation. Fetuses were extracted and then euthanized by hypothermia, followed by decapitation.

### Routine histology

Hematoxylin & eosin (H&E) staining was performed as reported previously [11]. In brief, E13.5 and E15.5 embryos were fixed with 4% paraformaldehyde (PFA), embedded, and cut into 12 micron frozen sections. Brain or thoracic sections were rinsed with distilled water for 5 min and then stained with 1× Hematoxylin solution (Cat.# HHS32, Sigma, St. Louis, MO, USA) for 3 min. After acid differentiation, samples were incubated with Scott’s solution for 30 s, washed with water and ethanol, and stained with alcoholic Eosin Y for 3 min. Sections were then dehydrated with ethanol and xylene, and then mounted with cytoseal 60 (Richard-Allan). Images were obtained with an Axioskop microscope (Zeiss, Germany). Alcian blue and Alizarin red staining was performed using previously described methods [12]. In brief, embryos from wild-type and mutant mice were euthanized, skinned and eviscerated, then dehydrated in 95% ethanol overnight, and acetone overnight. The embryos were then stained with Alizarin red (0.005%) and Alcian blue (0.015%) in a solution containing ethanol, glacial acetic acid and water (60:5:35) at 37°C overnight. The stained embryos were then transferred to a 1% potassium hydroxide solution for 2 days to dissolve the soft tissue; the cleared skeletons were preserved in glycerol.

### Immunostaining

Immunostaining was performed according to previously described procedures [4,5]. Briefly, E13.5 and E15.5thoracic sections were rinsed twice with PBS and distilled water, and then dipped into antigen-retrieval buffer (10 mM citrate buffer, pH6.0). The sections were microwaved for 5–10 minutes, and then quickly cooled down with cold water for BrdU staining, or slowly cooled at room temperature for other antigen staining. The sections were blocked with 5% donkey serum in PBS, and then incubated overnight using the appropriate primary antibodies. The following antibodies with corresponding dilutions were used for tissue and cell staining: mouse anti-BrdU (1:100, Calbiochem), rabbit anti-Ki-67 monoclonal antibody (1:200, Epitomics, cat. 4203–1), mouse anti-FLNA (1:100, Santa Cruz sc-58764), mouse anti-Desmin (1:100, BD Bioscience 550626), and rabbit anti-caspase3 (1:100, Cell Signaling technology 9661). The following secondary antibodies were used: DyLight 488 and DyLight 594 donkey anti-mouse antibodies (1:200), and DyLight 488 and DyLight 594 donkey anti-rabbit antibodies (1:200) from Jackson Immunoresearch.

For BrdU labeling in vivo, BrdU injection was performed as previously described [4]. Briefly, E12.5 and E14.5 pregnant mice were injected once intra-peritoneally with BrdU (60mg/kg) and were sacrificed at a 24-hour interval. All the embryos were removed and fixed in 4% paraformaldehyde and processed for BrdU immunohistochemistry.

### Assay quantification and statistical analyses

The quantitative analysis was performed as described previously [4]. Briefly, to quantify the number of positively labeled cells on serial tissue sections, three homotopic sections in WT, null-FlnA, and null FlnA + Fmn2 littermate brains (n>3 animals per experimental variable) were used for staining. The number of proliferative cells from homologous representative areas with fixed surface areas were quantified in a double blinded fashion using NIH Image J from both experimental and control mice. Measurements of midline closure were quantified using the narrowest region of non-closure in the thoracic abdominal wall from gross anatomic photomicrographs with NIH Image J. Data are represented as the mean (n≥3) ± standard deviation. T value (two-tailed test) was calculated using formula T = ($\bar{X1}\text{-}\bar{X2}$)/√(S_1_^2^/n_1_-S_2_^2^/n_2_), P value was calculated by p value calculator online. Significance was determined as p value<0.05.

## Results and discussion

### Loss of FlnA and Fmn2 causes thoracoabdominal schisis and shortening of the gut

**Fig 1:**
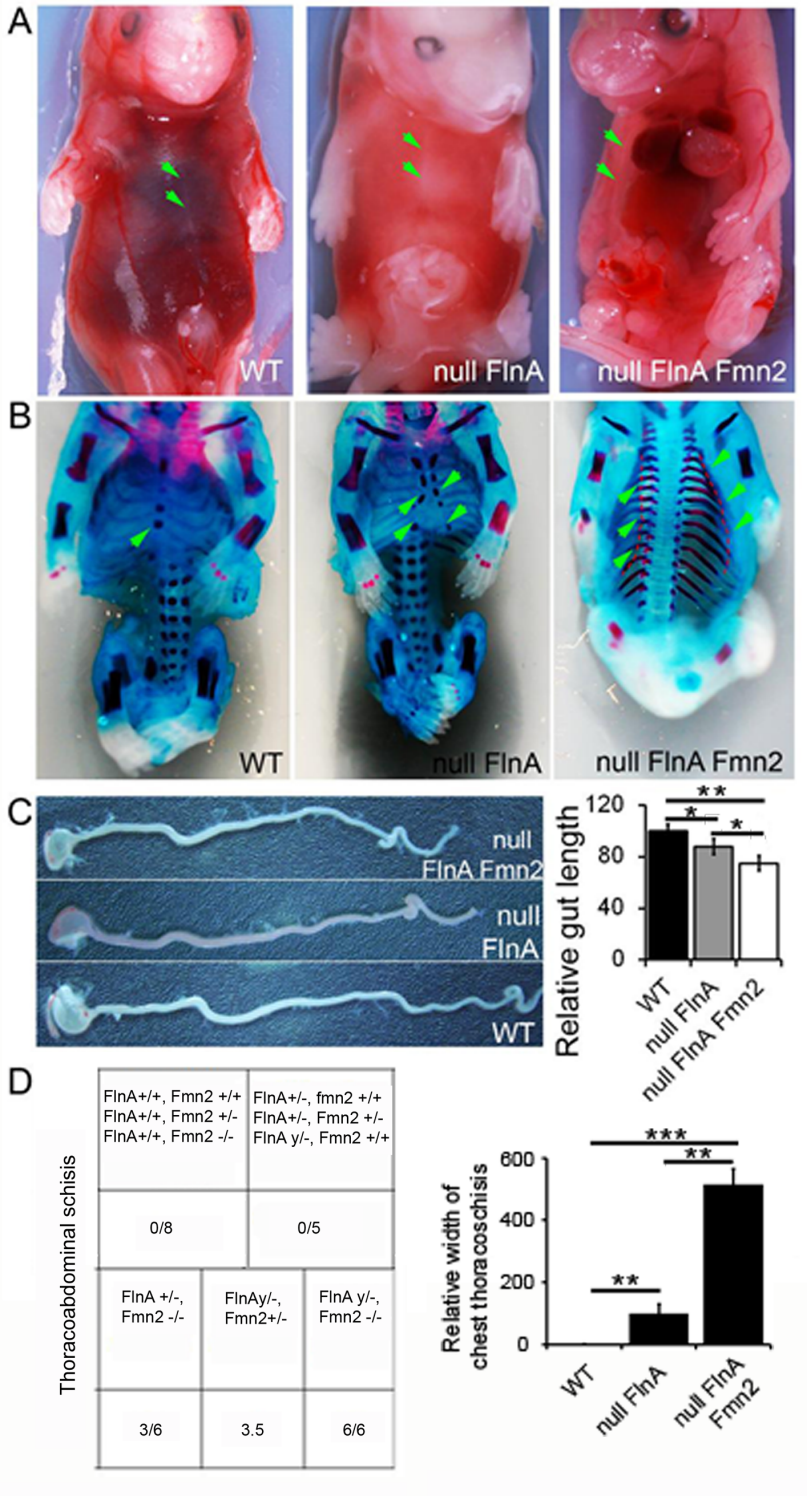
Loss of FlnA and Fmn2 causes a midline thoracoabdominal schisis, delayed bone formation and shortened gut. **A.** Gross anatomical photographs of FlnA+Fmn2 double knockout E15.5 embryos show a thoracoabdominal schisis that is characterized by midline extrusion of the heart and lungs in the ventral thorax, and the liver and intestines in the ventral abdomen. The overlying skin is also translucent and thinned. Noticeably, the ribcage/sternum (see green arrows) are localized to the lateral sides of the thorax in the null FlnA+Fmn2 mouse but was present in the ventral midline of WT or null FlnA mice (see arrows). **B.** Gross anatomical photographs of E15.5 mice following Alcian blue (cartilage) and Alizarin red (bone) staining shows a delay in development (ossification) of the ribcage in null FlnA+Fmn2 embryos, compared to null FlnA and WT age matched littermates. Midline closure of the sternum and ribs (green arrows) is completely absent in the double knockout mice, slightly delayed in the null FlnA mice and completed in the WT mice. The green arrows and dashed red lines in the null FlnA+Fmn2 mice indicate the contour of the sternum/ribs as they progress toward the midline. **C.** The intestines of null FlnA and null FlnA+Fmn2 embryos are shorter than that seen in WT embryos (WT vs null FlnA * = p <0.015, WT vs null FlnA+Fmn2 ** = p <0.004, null FlnA vs null FlnA+Fmn2 * = p <0.047). **D.** Findings of thoracoabdominal schisis formation in the null FlnA+Fmn2 mice during embryonic development are summarized in the table including the numbers of embryos examined. We also examined tens of null Fmn2 or null FlnA embryos from single gene-deleted mother. The results showed that loss of Fmn2 alone was insufficient to cause midline closure defects, and loss of FmnA caused a delay in midline closure as displayed in Fig 1B, but could lead to the formation of thoracoabdominal schisis. In contrast, approximately half of the heterozygous FlnA and Fmn2 embryos (3/6) or null FlnA and heterozygous Fmn2 embryos (3/5) actually developed thoracoabdominal schisis with extrusion of the organs through the midline, suggesting the additional effect of FlnA and Fmn2 functions on formation of thoracoabdominal schisis. Finally, all the null FlnA and Fmn2 embryos examined (6/6) developed thoracoabdominal schisis, contrast to only a midline closure delay for null FlnA embryos, indicating a significant difference in development of phenotypes between null FlnA alone and null FlnA and Fmn2 embryos. In addition, measurement of the schisis at the chest midline showed greater defects in midline closure in the double FlnA+Fmn2 null vs single FlnA null vs WT mice (graphically summarized, WT vs null FlnA: ** = p< 0.0017, null FlnA vs null FlnA+Fmn2: ** = p < 0.0025, WT vs null FlnA+Fmn2: *** = p < 0.0011).

To assess effects of FlnA and Fmn2 loss of function on embryonic development, we examined mice lacking expression of these two proteins at embryonic day 15.5 (E15.5). Whole animal preparations of WT, null FlnA and null FlnA+Fmn2 embryos show a midline closure defect with extrusion of the cardiopulmonary organs and abdominal organs (thoracoabdominal schisis) in FlnA+Fmn2 double knockout embryos as well as an abnormal localization of the ribcage and sternum at the lateral sides of the thorax (Fig 1A). Further analyses with Alcian blue (chondrocytes) and Alizarin red (bone) staining showed that the double null mice exhibited a delay in bone development in comparison to WT or null FlnA mice of the same age with decreased Alizarin red labeling in the appendicular bones and vertebrae (Fig 1B). No delay in midline closure or thoracoabdominal schisis with extrusion of organs at the midline was seen with Fmn2 null or heterozygous mice, expressing WT FlnA. A delay in midline closure but no thoracoabdominal schisis was appreciated in the FlnA null mice, expressing WT Fmn2. Approximately half of the FlnA null+Fmn2 heterozygous embryos and Fmn2 null+FlnA heterozygous embryos actually developed thoracoabdominal schisis with extrusion of the organs through the midline suggesting a FlnA and Fmn2 gene dose dependent penetrance (summarized in table in Fig 1D). Measurement of the schisis at the chest midline showed progressively worsened midline closure defects in the WT vs null FlnA vs null FlnA+Fmn2 mice (Fig 1D graph). Additionally, both null FlnA and null FlnA+Fmn2 embryos had underdeveloped intestines in comparison to that of WT embryos, in an increasingly severe fashion (Fig 1C).These observations suggest that while FlnA plays a more significant role in development of these phenotype with an added contribution through Fmn2, loss of both proteins are necessary to generate the fully penetrant thoracoabdominal schisis phenotype.

### Loss of FlnA and Fmn2 causes developmental defects in the muscles and heart

**Fig 2:**
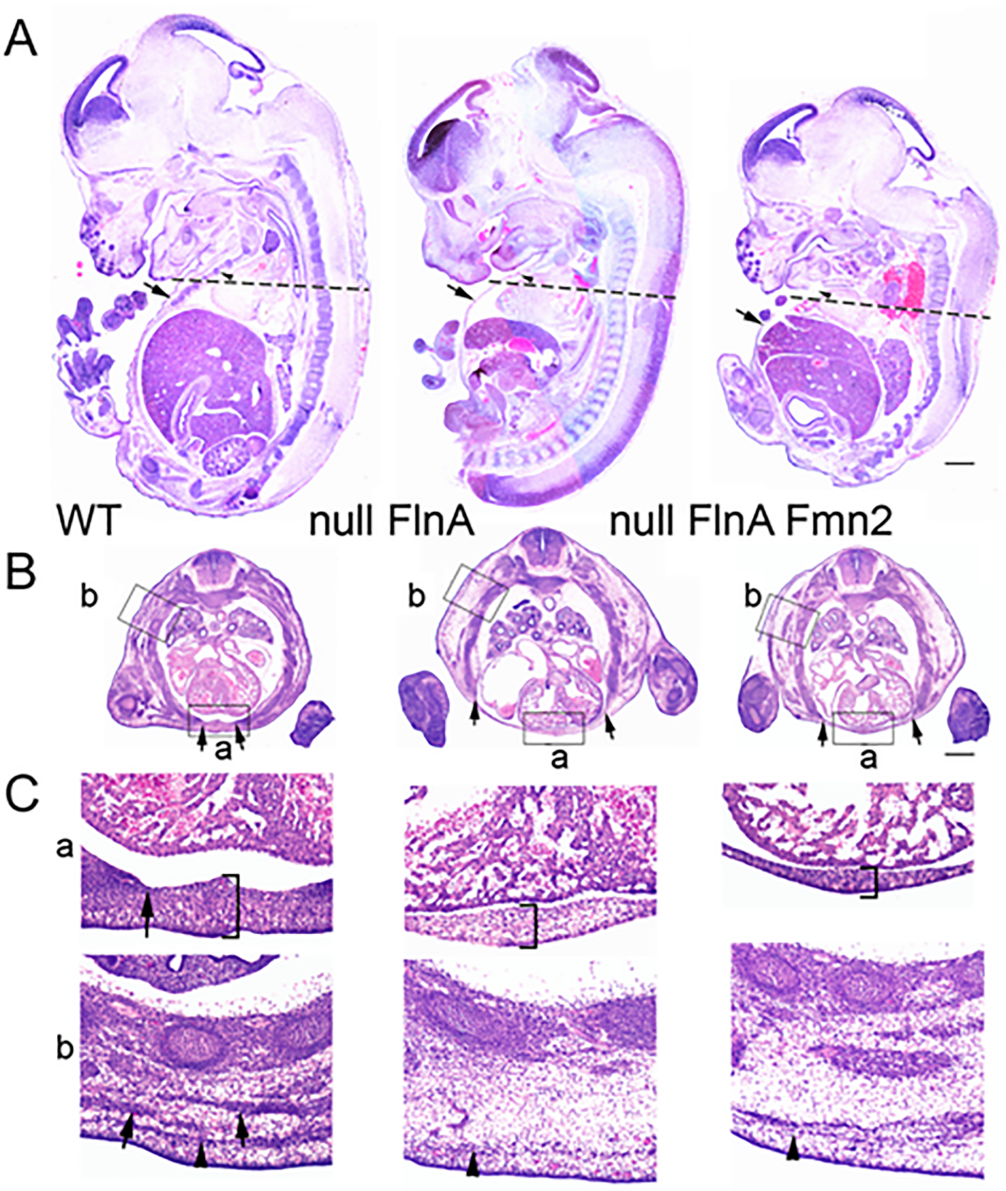
Loss of FlnA and Fmn2 leads to smaller body size, thinned muscle, and skeletal wall, as well as defects in cardiac valves. **A.** Brightfield photomicrographs of sagittal sections of E13.5 mouse embryos stained with hematoxylin and eosin (H&E) demonstrate the smaller body size and thinned or unformed ventral body wall (arrows) in the null FlnA and null FlnA+Fmn2 embryos. **B.** H&E staining of transverse whole body sections corresponding to the dashed lines in (A) shows severe developmental defects in the muscle/ribs along the lateral thoracic and ventral body wall. **C.** Higher magnification photomicrographs corresponding to boxed area (a) in panel B capture the thinned ventral body wall (bracket) in the double knockout mice. In the age-matched WT mice, the ventral wall is considerably thicker and the rib/sternum (arrow) has migrated near the midline. Higher magnification photomicrographs corresponding to the boxed area (b) in panel B display a severe defect in thoracic muscle formation in null FlnA, and null FlnA+Fmn2 embryos (arrows marked muscles). Scale bar = 500 μm.

To gain further insight into the developmental effects of FlnA and Fmn2 loss of function, we analyzed sagittal and transverse sections of WT, null FlnA, and null FlnA+Fmn2 embryos. The null FlnA embryos were smaller in body size than the WT embryos, while the null FlnA+Fmn2 embryos were comparatively the smallest. By using a sagittal section of the embryos, hematoxylin and eosin (H&E) staining revealed underdeveloped ventral body wall in null FlnA and null FlnA+Fmn2 embryos (Fig 2A). Defects could also be found in the muscle and ribs of null FlnA and null FlnA+Fmn2 embryo coronal sections (Fig 2B). A closer look at the ventral body wall of the age-matched WT, null FlnA, and null FlnA+Fmn2 mice reveals that the null FlnA mice have a thinner ventral body wall than WT mice as well as thoracic muscle formation defects; while the ventral body wall is even thinner and defects are more severe in null FlnA+Fmn2 mice (Fig 2C).

### Loss of FlnA and Fmn2 decreased cell proliferation in ribcage and ventral midline

**Fig 3:**
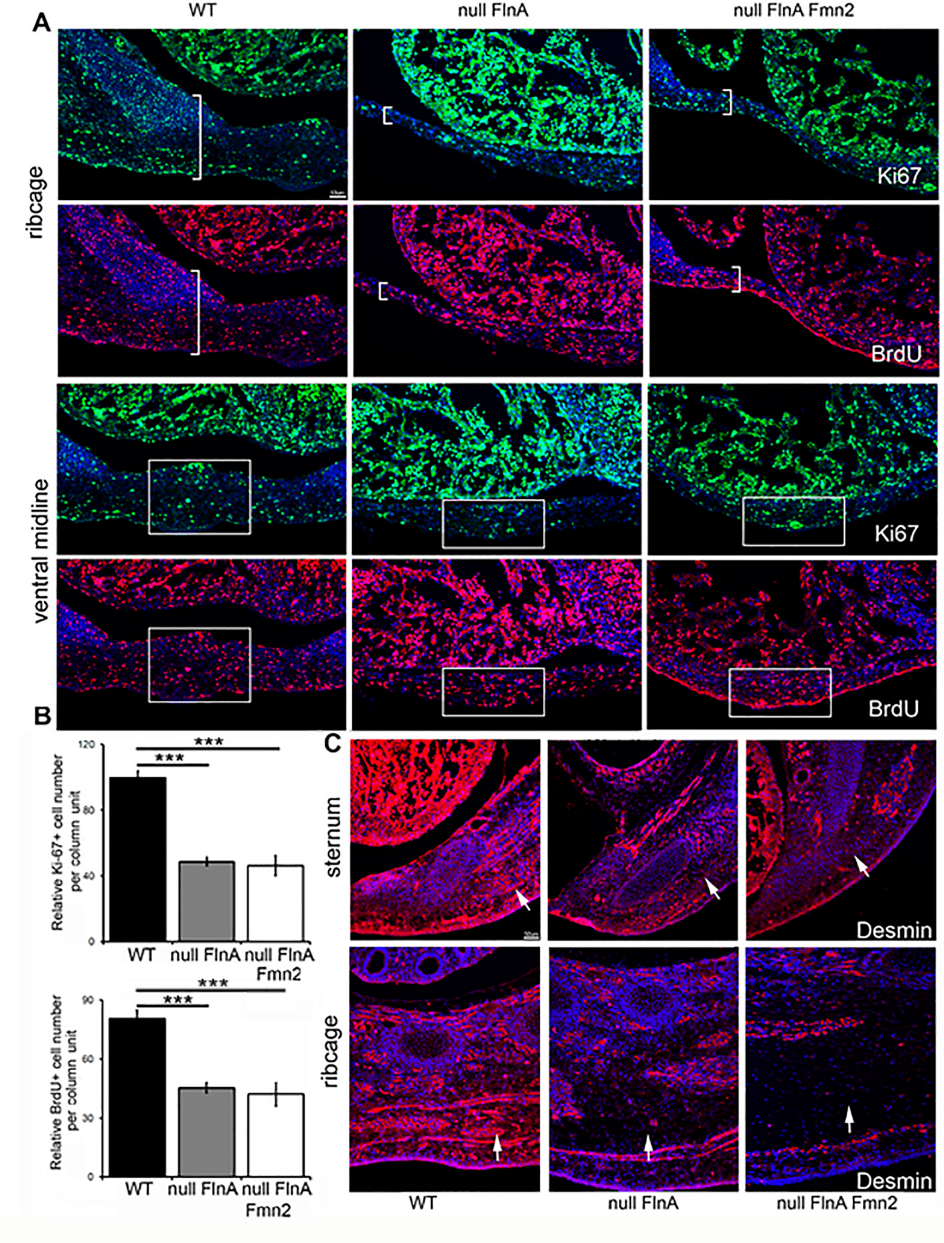
Loss of FlnA and Fmn2 leads to a defect in cell proliferation along the ventral thoracic wall. **A.** Fluorescent photomicrographs of immunostaining performed on transverse sections of E13.5 mouse thorax for proliferation markers. Ki-67 (a marker for cells in the cell cycle) and BrdU (a marker labeling cells entering into S phase) revealed a significant decrease in the number of proliferating (Ki-67^+^ or BrdU^+^) cells per unit area in the ribcage and ventral midline of the null FlnA and null FlnA+Fmn2 embryos compared to control WT embryo. Brackets mark the thickness of the ribcage, whereas the rectangular box delineates the thickness of the ventral midline. **B.** The number of BrdU or Ki67 positive cells in the ventral midline of null FlnA and null FlnA+Fmn2 embryos was decreased by more than 50% in comparison with those in WT embryo (WT vs null FlnA *** = p <0.0002, WT vs null FlnA+Fmn2 *** = p <0.0008). **C.** Immunostaining of E13.5 mouse ribcage and sternum for desmin, a mesenchymal and muscle cell marker reveals a reduction in the numbers of mesenchymal-derived cells in null FlnA and null FlnA+Fmn2 embryos (arrows). Nuclei are counterstained with DAPI (blue color) in all the images. Three homotopic sections were examined from n>3 littermate mice per experimental variable. Scale bars = 50 μm.

Delays in skeletal development can be due to altered proliferation. We therefore used fluorescent immunostaining on transverse sections of the thorax of WT, null FlnA, and null FlnA+Fmn2 mice and stained for two proliferation markers, Ki-67 (marker for cells that are in the cell cycle) and BrdU (marker for cells that have entered into S phase at the time of BrdU labeling). We observed an approximately 50% reduction in the number of Ki-67+ or Brdu+ cells within the thoracic wall of the null FlnA and null FlnA+Fmn2 compared to age-matched control, although no significant difference was seen on comparison of proliferative rates between the FlnA versus FlnA+Fmn2 nulls (Fig 3A and 3B). These findings likely reflect a reduction in proliferation leading to the observed midline defects. In addition, staining with a mesenchymal/myofibroblast marker, desmin, revealed an overall decrease in the number of mesenchymal derived cells in the sternum and ribcage (Fig 3C), which may indicate a detrimental effect in cell differentiation.

### Thoracoabdominal schisis in double null mice is not caused by programmed cell death

**Fig 4:**
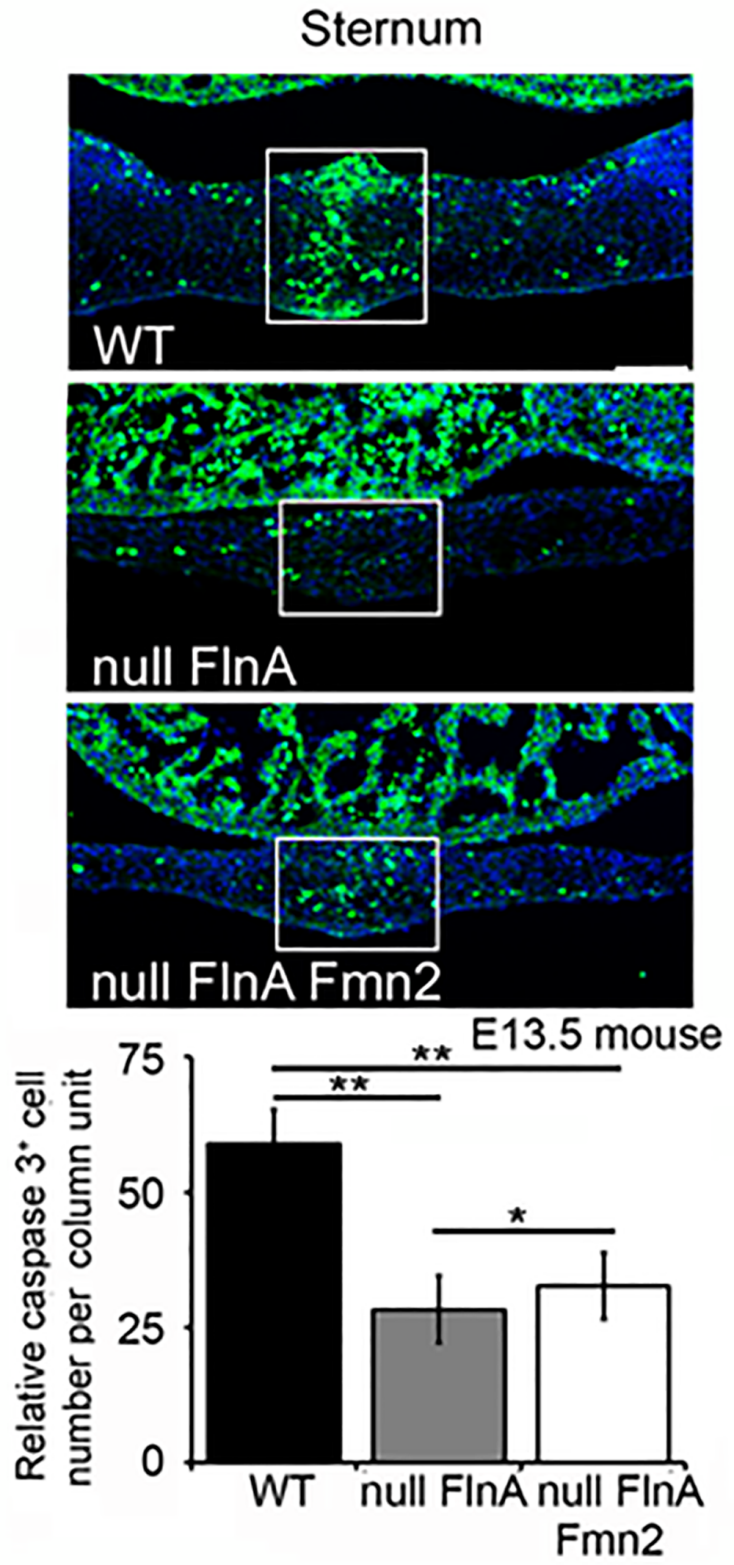
Programmed cell death does not cause the thoracoabdominal schisis seen in the FlnA+Fmn2 double null mice. Fluorescent photomicrographs show decreased rates of programmed cell death (fluorescein) between WT vs null FlnA, and WT vs null FlnA+Fmn2 cortex within the mouse sternum (WT vs null FlnA ** = p < 0.00012, Wt vs null FlnA+Fmn2 ** = p < 0.0004, null FlnA vs null FlnA+Fmn2 * = p < 0.05). Immunostaining was performed on E13.5 ventral walls of the thorax (lower panels) for caspase 3, an apoptotic marker. Nuclei are counterstained with DAPI (blue color). Three homotopic sections were examined from n>3 littermate mice per experimental variable. Scale bars = 50 μm.

Although the loss of FlnA and Fmn2 impairs cell proliferation, increased cell death could also contribute to the phenotype. Through immunostaining of the sternum in WT, null FlnA, null FlnA+Fmn2 mice we observed a significant decrease in the programmed cell death rate (caspase 9 labeling) between WT and either genetically engineered mice. Moreover, increased cell death was seen in the null FlnA + Fmn2 mice relative to the null FlnA alone. (Fig 4).

## Discussion

In addition to their known roles in brain development [4,9,13–15], the current studies show that actin-binding FlnA and actin-nucleating Fmn2 influence the development of various organ systems, including the gut, muscle, and skeleton. Moreover, their loss of function appears to impair the proliferation of presumptive mesenchymal cells which give rise to the myoblasts chondrocytes and osteoblasts found in thethe skeletal and thoracoabdominal wall. The previously noted reduction in brain size and shortening of the gut (endodermal derivation), as well as the thinning of the skin (ectodermal derivation), suggest that these proteins influence the proliferation and cell death of a wide array of progenitor types. Presumably, disruption in actin dependent vesicle trafficking plays a primary role for the observed phenotypes.

Our prior studies in the central nervous system provide a framework with which to potentially understand the mechanisms underlying the changes in cell proliferation. FlnA binds various neural cell membrane receptors including integrins and LRP6, which mediate activation of FlnA-binding RhoGTPases such as RhoA [7]. FlnA also binds to Fmn2, and these two proteins interact to mediate stability and endocytosis of Wnt associated receptors such as Lrp6 at the cell membrane [9]. Fmn2, however, also exhibits N to C terminal binding which leads to an autoinhibition, as this closed loop prevents interaction of the Fmn2 formin homology (FH1/2) domains with other molecules. Activated RhoA competitively inhibits this N-C terminal binding to open the FH1/2 domains for interactions with ubiquitin ligases. We recently showed binding of Fmn2 to the E3 ubiquitin ligase Smurf2. Smurf2 regulates degradation of Disheveled in the canonical wnt pathway to mediate degradation of beta catenin and thereby direct neural proliferation. Similar players including Lrp and Wnt have been implicated in bone development and intestinal stem cells [16,17]. The Wnt/β-catenin pathway also regulates cardiac valve formation [18]. In this respect, analogous molecular pathways seen with the involvement of FlnA and Fmn2 in regulating neural proliferation are likely involved in the control of cell proliferation in these other tissues and organs.

Changes in programmed cell death also affect the FlnA and Fmn2 phenotype. We observed decreased programmed cell death in the FlnA or FlnA+Fmn2 knockout mice relative to control. While the mechanism giving rise to these differences are not known, this decline may merely relate to the decrease in the total number of cell generated with the reduced proliferative rate. Of note, we also see that the double FlnA+Fmn2 null shows greater rates or cell death relative to the FlnA null and this may in part account for the worsened severity in phenotype seen with the double null. Further studies would be necessary to try to understand how the filamin and formin interactions lead to this change.

Various combinations of filamins, formins, and their interacting partners allow for the generation of specific temporal and spatial regulation—and the potential to give rise to various developmental anomalies. Filamins are comprised of three homologous proteins—Filamin A, B, C. Human mutations in FLNB have been associated primarily with skeletal anomalies, whereas FLNC mutations cause myopathies [19,20]. In this study, FlnA appears to contribute to diverse developmental defects, consistent with its broad expression. FlnA also binds over 30 receptors and intracellular constituents (Yang and Walsh, review), further extending the various combinations of interactions that could affect developmental processes. We and others have shown that Flns can bind various RhoGTPases and that FlnA and FlnB exist as heterodimers to mediate Rho GTPase activity [7,21]. Consistent with this interdependence, loss of FlnA leads to enhanced expression of FlnB in chondrocytes [7]. In a similar fashion, some 15 different formin proteins exist and have been categorized into 7 subgroups [22]. Formin activation can be dependent on RhoGTPases and regulate endosomal vesicle trafficking [9]. With the release of this autoinhibition, formins can bind to various ubiquitin ligases to mediate lysosomal degradation. Collectively, these many interactive proteins allow for innumerable combinations with which to finely regulate cell type and temporal specific developmental processes.

The similarities in phenotype seen in PoC and loss of FlnA and Fmn2 function may suggest some common mechanistic pathway. PoC has been associated with disruption of the homeobox b4 gene (*Hoxb-4*) in mice[3] Loss of Hoxb-4 function leads to a defective morphogenesis of the sternum in mice. [3]. As transcription factors, homeobox genes regulate the localization and expression of a wide array of targeted genes in coordinated fashion to direct the formation of many body structures during early embryonic development. Vesicle trafficking is essential to the transportation of various molecules within the cell and between the cell and its environment. This process indirectly regulates the location and expression of various cell determinants that influence developmental processes such as progenitor proliferation, migration, and differentiation [4,9,13]. Disruption of filamins and formins could therefore alter the localization and degradation of various actin-binding proteins [23], and thereby disrupt potential cell/tissue polarization (as seen with homeobox genes such as *Hoxb4*) that might lead to features seen in PoC. Interestingly, similar to the FlnA+Fmn2 mice (dependent on homo and heterozygosity) there can be incomplete penetrance and variable expressivity in the Hoxb-4 null mice, when assayed in the hybrid genetic background. The sternum defect is completely penetrant in the inbred Hoxb-4 background as it is in the double null FlnA+Fmn2 mice. These observations suggest potentially that disrupting the localization or expression patterns of specific proteins–either through transcription factor regulation or vesicle trafficking can lead to the PoC.

Much of our understanding of the Fln and Fmn actin associated proteins have focused on their roles in development- and this stems from the very fact that human mutations in these genes give rise to congenital disorders [24,25]. However, FlnA has been implicated in various other diseases that occur later in life such as epilepsy, Alzheimer’s disease, and certain malignancies. The extent to which these FlnA and Fmn2- associated actin dependent vesicle trafficking play a role in the pathogenesis of these disorders, and whether targeting these pathways can prove useful for therapeutic intervention, remains to be seen.

## Conclusion

The mechanisms leading to the developmental malformation thoracoabdominal schisis are not well understood. Loss of the actin-binding FlnA and actin-nucleating Fmn2 collectively give rise to this midline defect. As seen in the brain where loss of FlnA and Fmn2 leads to microcephaly due to impaired neural progenitor proliferation, a similar decrease in cell proliferation rates is observed in the thoracic wall and contributes to the phenotype. These findings suggest that FlnA and Fmn2 likely play broad roles in progenitor development across multiple organ systems due to their known functions in regulating vesicle trafficking.
